# Male Accessory Glands of Blister Beetles and Cantharidin Release: A Comparative Ultrastructural Analysis

**DOI:** 10.3390/insects13020132

**Published:** 2022-01-26

**Authors:** Maurizio Muzzi, Emiliano Mancini, Emiliano Fratini, Manuela Cervelli, Tecla Gasperi, Paolo Mariottini, Tiziana Persichini, Marco Alberto Bologna, Andrea Di Giulio

**Affiliations:** 1 Department of Science, University Roma Tre, 00146 Rome, Italy; maurizio.muzzi@uniroma3.it (M.M.); emiliano.fratini@uniroma3.it (E.F.); manuela.cervelli@uniroma3.it (M.C.); tecla.gasperi@uniroma3.it (T.G.); paolo.mariottini@uniroma3.it (P.M.); tiziana.persichini@uniroma3.it (T.P.); marcoalberto.bologna@uniroma3.it (M.A.B.); 2 Laboratorio Interdipartimentale di Microscopia Elettronica (LIME), University Roma Tre, 00146 Rome, Italy; 3 Department of Biology and Biotechnology “C. Darwin”, “Sapienza” University of Rome, 00185 Rome, Italy; emiliano.mancini@uniroma1.it

**Keywords:** Meloidae, male reproductive system, accessory glands, *vas deferens*, electron microscopy, FIB/SEM

## Abstract

**Simple Summary:**

Meloidae, also called blister beetles, are known to actively produce cantharidin, a toxic terpene with a defensive function that is released externally by reflex bleeding, and that is also stored in large quantities in the male accessory glands. These glands are involved in the transfer of terpene from males to females, which receive cantharidin via spermatophores as a nuptial gift to be used for their own protection and that of the eggs. However, it is still debated whether the male accessory glands can actively produce the terpene or if they only mediate its transfer, since neither the cantharidin-producing organ nor the metabolic pathway are known to date. The focus of the work is to analyze comparatively the accessory glands of males in representative Meloidae species to provide morphological evidences that can contribute to this debate. The results highlight the complexity of the accessory gland system, consisting of three different types of glands that are highly variable between species with the exception of one, which remains conserved even in independent phyletic lines. This gland is a good candidate for hypothesizing a direct role in cantharidin production and/or concentration.

**Abstract:**

Members of the family Meloidae are known to produce cantharidin, a highly toxic monoterpene found in their hemolymph and exuded as droplets capable of deterring many predators. As a nuptial gift, males transfer large amounts of cantharidin to females via a spermatophore, which is formed by specific accessory glands containing high concentrations of this terpene. Using light, electron and ion beam microscopy, the ultrastructural features of the three pairs of male accessory glands as well as the glandular part of the *vasa deferentia* were comparatively investigated in seven species of blister beetles belonging to five different tribes and two subfamilies. All gland pairs examined share common features such as mesodermal derivation, the presence of muscle sheath, a developed rough endoplasmic reticulum, abundant mitochondria, secretory vesicles, and microvillated apical membranes. Within the same species, glands exhibit distinctive features, suggesting that each pair is responsible for the formation of a specific substance. The *vasa deferentia*, while showing many similarities within the family, often exhibit features unique to each of the individual species investigated, whereas the accessory glands of the first and second pairs display the highest degree of ultrastructural variability. A comparison across the species shows an interesting constancy limited to ultrastructural features in the third pair of accessory glands. The similarities and differences among the species are discussed in the light of the available literature and in relation to the potential role that blister beetles’ male accessory glands could play in the storage and management of cantharidin.

## 1. Introduction

Meloidae is a beetle family that includes 130 genera and about 3000 species distributed in temperate and tropical regions all over the world [1,2,3,4,5,6]. The members of this family represent a paradigmatic example of hypermetamorphosis, showing a succession of larval instars that are functionally and morphologically different from each other [7,8,9,10]. These striking differences are mostly aimed at reaching and exploiting the host’s resources, in the case of parasitic species of Apoidea, or at finding grasshoppers *Ootecae* to feed on the eggs, for the predatory species of Orthoptera, such as several *Mylabrini* and *Epicauta* Dejean, 1834 species that exploit locust egg-pods in their juvenile stages.

As suggested by their common name, blister beetles, these insects are well known and popularly recognized for having a blistering action, which is due to their ability to synthesize and internally store high amounts of cantharidin [11,12,13,14], a monoterpene which is highly toxic to many animals and that blister beetles release externally through reflex bleeding as a hemolymphatic exudate. This latter is emitted in the form of yellow, oily droplets from the joints of legs and antennae in order to obtain an effective chemical protection against a large number of natural predators [15,16].

It is noteworthy that cantharidin is not exclusively produced by blister beetles but is also synthesized ex-novo by beetles belonging to the family Oedemeridae [17,18]. Nevertheless, it is mainly members of the Meloidae family that have been widely used in the traditional medicines and pharmacology of different cultures and countries, e.g., pre-Columbian civilizations, the Roman Empire or in ancient China over two thousand years ago [19,20]. Indeed, despite its high toxicity and the wide range of adverse effects following its ingestion, cantharidin extracted from these insects has been popular not only for its blistering effects, but also as an antiphlogistic and as a supposed aphrodisiac [21,22,23]. Even today, this terpene is employed, albeit to a limited extent and under strict medical supervision, in the dermatological field for the topical treatment of warts [21,24]. In addition, together with its derivatives, cantharidin is being considered as an alternative compound for anti-cancer treatments [25,26,27,28,29].

Although it is well established that the hemolymph of both male and female blister beetles contains cantharidin [13,14], it is still a matter of debate whether females are able to actively and equally efficiently synthesize this compound as males. This uncertainty arises from the fact that males are reported to have a higher concentration of cantharidin than females [30,31,32,33] and, more importantly, a high concentration of cantharidin in their accessory glands [11,34,35,36], most of which is passed to the female during copulation via the spermatophore [37,38,39,40,41]. Given the paucity of laboratory studies on virgins [11,42,43,44], it remains unclear whether adult females also have an equal ability to synthesize cantharidin compared to males or whether the high concentration in their hemolymph is due to previous mating. Interestingly, the tissues responsible for cantharidin production have not yet been identified in males either, and while the high content of terpene might suggest an active involvement of the reproductive system’s accessory glands in cantharidin synthesis, other studies indicate that these probably only serve as reservoirs [45,46] and that the tissues responsible for such synthesis are others, of which fat bodies have been suggested [45].

In blister beetles there are three pairs of large male accessory glands and a pair of well-developed *vasa deferentia* with glandular function [22,23]. In the past, these structures of the male reproductive system have been the focus of several comparative works that have illustrated their general structure [22,47,48,49,50] and, in some cases, also provided some histological details, as in the case of *Lytta nuttalli* Say, 1824 [51,52].

Ultrastructural information on the male accessory glands has only recently been provided for the species *Meloe proscarabaeus* Linnaeus, 1758 [53], showing that different pairs of glands have distinct ultrastructural features and that the epithelial cells of the third pair of glands seem to have an extensive exchange of material with the hemolymph, leading to the hypothesis that they might be involved in the transport, concentration and/or processing of cantharidin that is associated with the hemolymph.

The aim of the present work is to comparatively analyze the morphology and ultrastructure of the male accessory glands of different species belonging to six genera, representative of the main subfamilies (Meloinae and Nemognatinae) and tribes of blister beetles (Mylabrini, Lyttini, Epicautini, Cerocomini, Nemognatini). The results of this analysis will be compared with those available for the species *Meloe proscarabaeus,* representative of the tribe Meloini. In particular, the objective is to investigate which morphological and ultrastructural features of the male accessory glands are retained among the different species and whether the eventual differences reflect phylogenetic relationships.

## 2. Material and Methods

### 2.1. Collection of Specimens

**Figure 1:**
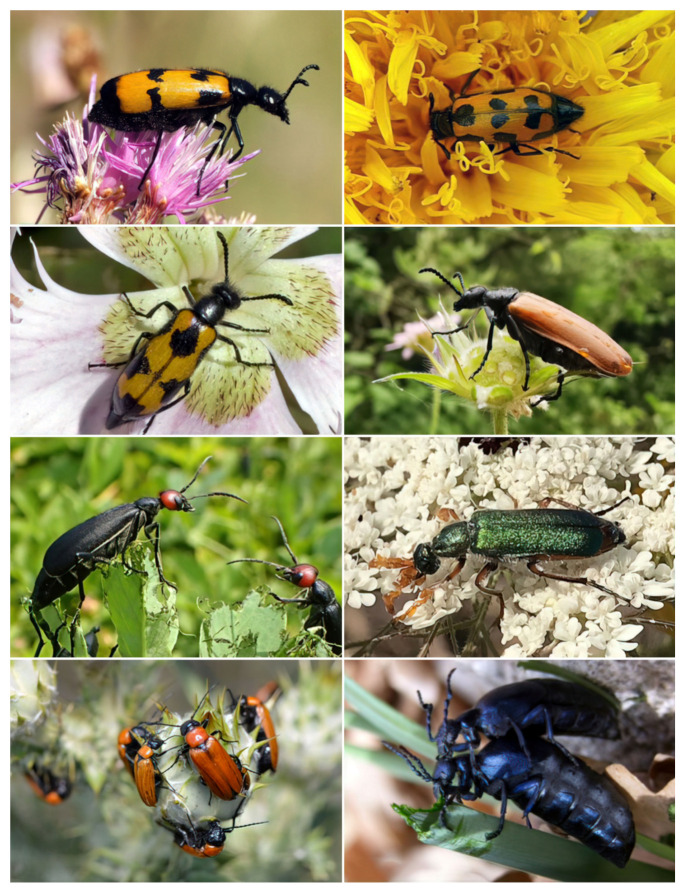
Photos of the different species of blister beetles analyzed comparatively in this work. From top to bottom and from left to right: *Mylabris variabilis*; *M. flexuosa*; *M. pusilla*; *Lydus trimaculatus*; *Epicauta rufidorsum*; *Cerocoma schreberi*; *Zonitis immaculata* and *Meloe proscarabaeus*.

Male Meloidae (listed below) were captured at different localities in northern and central Italy (Figure 1). Specimens were hand-picked during the warmest hours of the day, while feeding on flowers (Asteraceae, Dipsacaceae or Fabaceae, depending on the species) or during courtship behavior. Beetles were identified using taxonomic keys [23] and kept alive for a maximum of 6 days by placing them in fauna-boxes containing a moist substrate of coconut fiber and fresh flowers and/or apple slices as daily food.

*Lydus trimaculatus* Fabricius, 1775, 6 specimens, Lazio, Roma, Tolfa Mts., June 2019;

*Mylabris variabilis* (Pallas, 1781), 6 specimens, Lazio, Roma, Tolfa Mts., July 2019;

*Mylabris pusilla* Olivier, 1811, 4 specimens, Abruzzo, Aquila, Rocca di Mezzo, July 2019;

*Mylabris flexuosa*, Olivier, 1811, 4 specimens, Abruzzo, Aquila, Rocca di cambio, July 2019;

*Epicauta rufidorsum* (Goeze, 1777),3 specimens, Lombardia, Mantova, Marmirolo, August 2019;

*Cerocoma schreberi* Fabricius, 1781, 3 specimens, Lazio, Roma, Maccarese, May 2020;

*Zonitis immaculata* (Olivier, 1789), 3 specimens, Lazio, Roma, Maccarese, June 2020.

### 2.2. Light Microscopy

Two specimens of *Mylabris variabilis* and two of *Lydus trimaculatus* were anesthetized and euthanized with CO_2_ and their reproductive systems were rapidly dissected in saline solution under a SZX51 stereo microscope (Olympus, Tokyo, Japan). Pictures of the dissected systems were acquired using an OM-D E-M5 digital camera (Olympus, Tokyo, Japan) mounted on an Axio Zoom V16 microscope (Carl Zeiss AG; Oberkochen, Germany). Following the electron microscopy preparation, reported in the next paragraph, the dissected tissue materials were also used to create schematic diagrams of the male accessory gland system of the analyzed species. The presented diagrams were drawn and refined using Adobe Illustrator CS6 (Adobe, San Jose, CA, USA).

### 2.3. Focused Ion Beam/Scanning Electron Microscopy (FIB/SEM)

Live blister beetle males were euthanized with CO_2_ prior to dissection: their abdomens were removed and immediately submerged in cacodylate buffer 0.1 M (pH 7.4) in order to isolate the accessory glands after the removal of ventrites. Each gland was cut in smaller pieces to facilitate the subsequent fixation and staining processes. The small pieces of glandular tissues were immersed in Karnovsky’s solution for 12 h at 4 °C, rinsed four times in cacodylate buffer 0.1 M (pH 7.4) for 15 min, post-fixed in 1% osmium tetroxide in cacodylate buffer 0.1 M (pH 7.4) for 2 h at 4 °C, and en-bloc stained with 2% aqueous uranyl acetate. Subsequently, the samples were dehydrated in a graded ethanol series (70%, 85%, 95%, 30 min each and 100% for 2 h), embedded in epoxy resin and finally polymerized for 72 h at 60 °C.

In order to acquire ultrastructural information, the resin-embedded samples were processed in two different ways. The first involved cutting the resin blocks containing the tissues with a diamond knife (Diatome Ltd., Bienne, Switzerland) on an Ultracut T ultramicrotome (Leica Microsystems, Vienna, Austria). The resulting ultrathin sections were collected on TEM grids and observed using the STEM detector of a Dual Beam (FIB/SEM) Helios Nanolab 600 (FEI Company, Hillsboro, FL, USA) at the electron microscopy laboratory of the Roma Tre University (LIME, Rome, Italy). Alternatively, resin-embedded samples were cut into sequential slices, 15–20 μm thick, using the aforementioned ultramicrotome equipped with a glass knife. Thick slices were attached to aluminum stubs with a conductive adhesive carbon disk, sputtered with a thin layer (30 nm) of gold in a K550 sputter coater (Emithech, Kent, UK), and analyzed with FIB/SEM following the ‘Slice & Mill’ method [54], which allows milling of regions of interest, by using ion beam, and imaging of ultrastructural details present on the freshly milled surface by detecting secondary electrons.

## 3. Results

In this section, the gross morphology of the system will be presented in a more generalized form, giving particular attention to those features that are common to all the analyzed genera. With regard to the ultrastructural features of the different glandular tissues, the genus *Mylabris* Fabricius, 1775, will be the first to be described in detail, with an emphasis on the species *M. variabilis* that exhibits glands with intermediate characteristics. Subsequently, the ultrastructural features of the species belonging to the other genera will be comparatively presented, in order to highlight differences and similarities.

### 3.1. Gross Morphology of Male Accessory Glands and Vasa Deferentia

**Figure 2:**
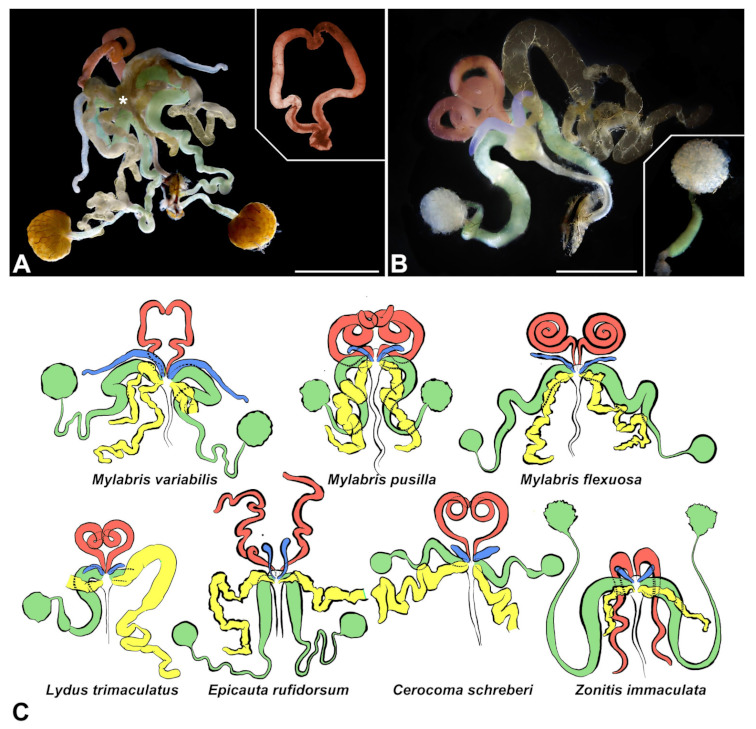
General structure of the male reproductive systems of the investigated Meloidae species. The different components are colored using specific colors: First pair of accessory glands in red, second pair of accessory glands in light blue, third pair of accessory glands in yellow and internal reproductive system in green. (**A**) Light micrograph of the dissected system of *M. variabilis* in ventral view; inset displaying the first pair of glands distended and separated from the reproductive system. Asterisk indicates the bifurcation of the third pair of glands, located near the point of insertion. (**B**) Light micrograph of the dissected system of *L. trimaculatus* in ventral view, the gland of the third pair on the left and the *vas deferens* on the right have been purposely removed; inset presenting a close-up of the expanded horn-like region of the *vas deferens*. (**C**) Schematic drawings of the systems of the different species illustrated in ventral view (diagrams are not in scale). The same colors were used as previously presented; ejaculatory duct was not colored. Scale bars: (**A**) = 1 mm; (**B**) = 2 mm.

The male internal reproductive system, although showing a moderate degree of variability in the blister beetles analyzed here, is always composed of a pair of testes and two *vasa deferentia* which, together with three pairs of accessory glands of different shapes, discharge their luminal contents into a more or less expanded area above the ejaculatory duct (Figure 2). The ejaculatory duct is the only component of ectodermal origin, while all the others are of a mesodermal derivation; it is therefore the only part of the internal reproductive system presenting a cuticular lining.

Each *vas deferens* is connected to a spherical or ovoid testis, made up of radially arranged and tightly packed follicles (Figure 2A,B). In the vicinity of the testis, the *vas deferens* appears as a short tube of a small diameter serving as a seminal vesicle, which may or may not present a distinct horn-shaped region (Figure 2A,B). The seminal vesicle is then followed by a very long and well-developed glandular region, characterized by a serpentine course and an increasing diameter that reaches its maximum near its point of insertion in the expanded region above the ejaculatory duct.

The accessory glands of the first pair (Figure 2) appear as tubular structures with a blind anterior end that might join that of the corresponding symmetrical gland; their diameter is usually maximum in the central region while it decreases towards the blind apical area and their point of insertion, which is in the expanded area above the ejaculatory duct, antero-dorsally to the *vasa deferentia*. Glands of the first pair can present a different degree of coiling depending on the species analyzed. In some of them the glands are organized as tightly coiled spirals, while in others, they are only slightly curved with some regions showing a sharper folding (Figure 2C).

The accessory glands of the second pair are tubular structures with a blind apex and a more or less constant diameter. They usually do not show a particular degree of flexion or coiling, except for a possible minimal distal curl observed in some species. Their relative length is variable according to the species analyzed, ranging from a more common condition in which it is very short, to an infrequent condition of markedly long glands (Figure 2C). Their insertion is always postero-ventral in the mesodermal region overlying the ejaculatory duct.

The third pair of accessory glands is always much more fragile and less turgid compared to the other pairs; these irregularly shaped glands always show a great development in length and a marked sinuosity that leads them to be often entangled with the other components of the reproductive system (Figure 2C). Distinctive constriction points along the gland are commonly present in some species, contributing to their irregular shape. The main difference found at the level of these structures in the different species analyzed concerns the possible ramification of the gland. In fact, while they appear unbranched in most of the species analyzed, in *M. variabilis* these glands show a clear bifurcation near the insertion point (Figure 2A), which is always postero-ventral at the level of the expanded region surmounting the ejaculatory duct.

### 3.2. Ultrastructural Features of Accessory Glands and Vasa Deferentia in Mylabris (Meloinae, Mylabrini)

**Figure 3:**
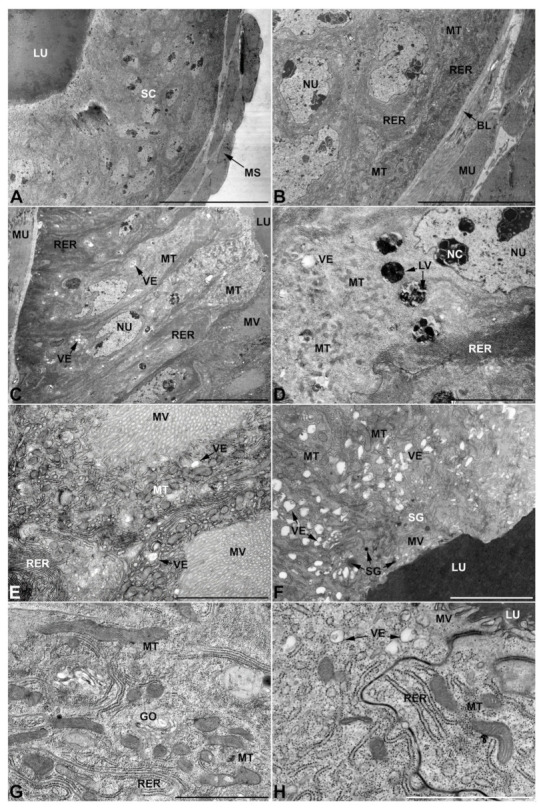
Ultrastructure of the first pair of male accessory glands in *M. variabilis*. (**A**) Cross section of the gland showing the epithelium pseudostratification, central lumen containing the secretions and a developed muscular sheath encasing the cells. (**B**) Close-up of the former micrograph showing muscles and basal regions of the cells with indented nuclei, mitochondria and developed rough endoplasmic reticulum stacks. (**C**) Longitudinal section of the glands showing polygonal appearance of the cells and apical microvillated plasma membrane. (**D**) Micrograph towards the cell apex depicting cytoplasm with numerous mitochondria, flattened rough endoplasmic reticulum stacks and different vesicles. (**E**) Plasma membrane developing an abundant network of crowded and overlapping microvilli. Note the presence of numerous mitochondria. (**F**) Micrograph of the interface between lumen and secretory cells showing numerous lucid vesicles and a few small secretory granules near the microvilli. (**G**) Close up of the cytoplasm illustrating slender mitochondrial morphology, small Golgi complexes, flattened rough endoplasmic reticulum and a pale inclusion. (**H**) Detail of the apical region showing connections between two adjacent cells, small electrolucent vesicles, endoplasmic reticulum, and free ribosomes. BL, basal lamina; GO, Golgi complex; LU, lumen; LV, large vesicles; MU, muscles; MS, muscular sheath; MT, mitochondria; MV, microvilli; NC, nucleolus; NU, nucleus; RER, rough endoplasmic reticulum; SC, secretory cells; SG, secretory granule; VE, vesicles. Scale bars: (**A**) = 30 μm; (**B**,**C**) = 10 μm; (**D**) = 5 μm; (**E**,**F**) = 3 μm; (**G**,**H**) = 2 μm.

**Figure 4:**
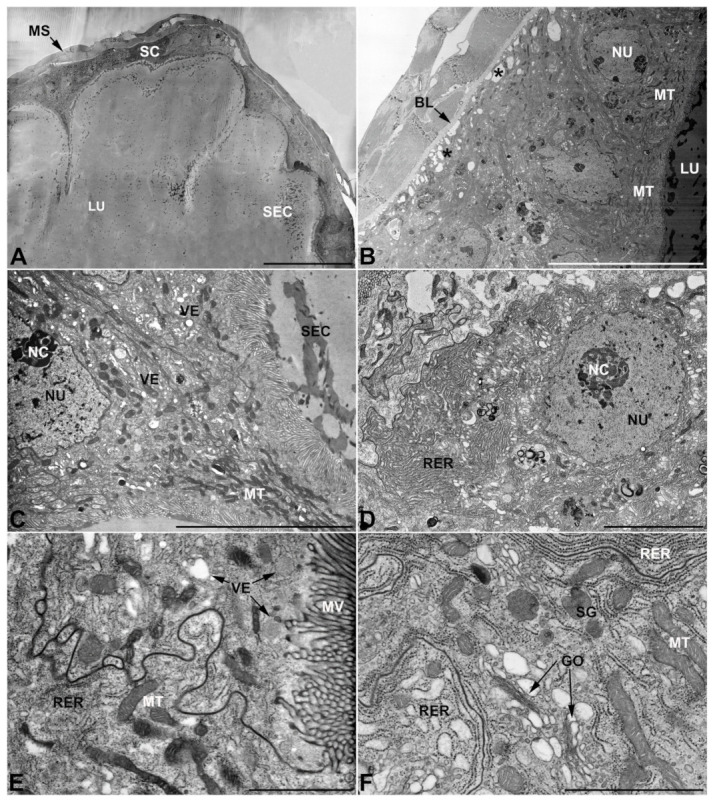
Ultrastructure of the second pair of male accessory glands in *M. variabilis*. (**A**) Cross section of the gland showing tissue pleats towards the lumen. (**B**) Micrograph showing the basal region of the cells with moderate folds of the basal membrane (indicated by the asterisks) and muscle fibers surrounding the secretory cells. (**C**) Close up at the level of the involution, displaying abundance of mitochondria and vesicles near the microvillated apical membrane. Some electrondense granules can also be observed in the cytoplasm. (**D**) Nucleus with obvious nucleolus surrounded by abundant flattened stacks of rough endoplasmic reticulum. (**E**) Apical region of the cell bearing microvilli. Note the sinuous contours of the lateral membranes of two neighboring cells, slender mitochondria and vesicles. (**F**) Close up showing flattened Golgi complex, thin mitochondria, dark granules and rough endoplasmic reticulum with both flattened and expanded cisterns. BL, basal lamina; GO, Golgi complex; LU, lumen; MS, muscular sheath; MT, mitochondria; MV, microvilli; NC, nucleolus; NU, nucleus; RER, rough endoplasmic reticulum; SC, secretory cells; SEC, secretions; VE, vesicles. Scale bars: (**A**) = 50 μm; (**B**) = 20 μm; (**C**) = 10 μm; (**D**) = 5 μm; (**E**,**F**) = 2 μm.

**Figure 5:**
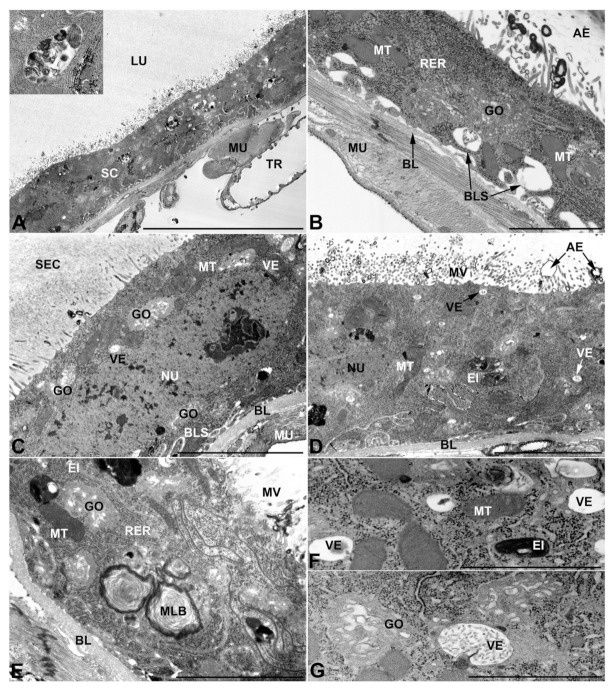
Ultrastructure of the third pair of male accessory glands in *M. variabilis*. (**A**) Cross section of the gland; note the particulate secretion inside the lumen. Inset depicting one of the autophagic vacuoles commonly encountered in these cells. (**B**) Micrograph of the secretory cell showing the lacunar system, Golgi complexes with very small cisternae, and flattened rough endoplasmic reticulum. Note the apical microvilli and the ampullaceous expansions. (**C**) Developed nucleus surrounded by small Golgi complex and mitochondria. In the lumen, the particulate secretion is clearly visible. (**D**) Micrograph displaying developed rough endoplasmic reticulum, Golgi with small dictyosomes, several vesicles (some of which are approaching the microvilli) and expanded region of the thin microvilli (**E**) Cell displaying multilamellar bodies, small Golgi complex, and electrondense inclusions. (**F**,**G**) Details of the vesicles, Golgi complex and mitochondria. AE, ampullaceous expansions; BL, basal lamina; BLS, basal lacunar system; EI, electrondense inclusion; GO, Golgi complex; LU, lumen; MLB, multilamellar body; MU, muscles; MT, mitochondria; MV, microvilli; NU, nucleus; RER, rough endoplasmic reticulum; SC, secretory cells; SEC, secretory products; TR, trachea; VE, vesicles. Scale bars: (**A**) = 20 μm; (**B**,**D**) = 5 μm; (**C**) = 5 μm; (**E**) = 4 μm; (**F**,**G**) = 2 μm.

**Figure 6:**
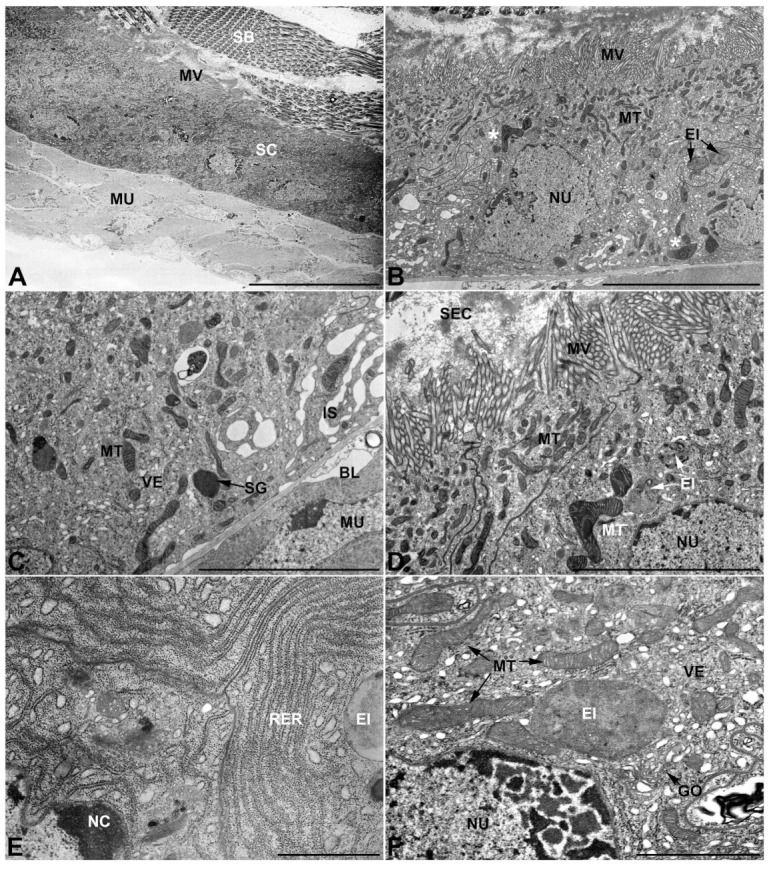
Ultrastructure of the *vasa deferentia* in *M. variabilis*. (**A**) Epithelium of the *vasa deferentia* encircled by developed muscles. Note the arrangement of the nuclei and the lumen containing bundles of spermatozoa. (**B**) Epithelium with abundant mitochondria and slightly indented nucleus, basally located. Note bigger mitochondria with angular shape (indicated by asterisks). (**C**) Basal region showing moderate intercellular spaces, polymorphic mitochondria and secretory granules. (**D**) Apical region of the cell with cytoplasm rich in mitochondria of various sizes and shapes, especially located near the microvilli. Note the fibrillar content of the lumen. (**E**) Detail of rough endoplasmic reticulum with slightly vacuolized cisternae. (**F**) Close up presenting Golgi complex, mitochondria, very small electronlucent vesicles and an electrondense inclusion. BL, basal lamina; EI, electrondense inclusions; GO, Golgi complex; IS, interecellular space; MU, muscles; MT, mitochondria; MV, microvilli; NC, nucleolus; NU, nucleus; RER, rough endoplasmic reticulum; SB, sperm bundles; SC, secretory cells; SEC, secretions; Sg, secretory granule; VE, vesicles. Scale bars: (**A**) = 20 μm; (**B**) = 10 μm; (**C**,**D**) = 5 μm; (**E**,**F**) = 2 μm.

- First pair of male accessory glands in *M. variabilis*. The first pair of male accessory has an outer muscular layer composed of an inner circular and outer longitudinal fibers enclosing a monolayered epithelium (Figure 3A–C). This latter circumscribes a large lumen filled with a homogeneous electrondense matrix in which irregularly shaped structures of higher electron density are immersed (Figure 3A). The mononucleated epithelial cells are about 35 μm high and lie on a basal lamina 0.4–0.6 μm thick (Figure 3B,C); they are tightly appressed to each other with a straight course along their medial regions (Figure 3C) and more sinuous contours towards the apex and base (Figure 3B–D). The apical plasma membrane is always characterized by the presence of many partially overlapping and crowded microvilli that face the glandular lumen (Figure 3E,F). The ovoid nucleus, approximately 7–10 μm in size, frequently shows indentations and one or two prominent nucleoli (Figure 3B–D). It is usually located in the basal region of the cell (Figure 3A,B), however, due to the pseudostratified organization of this epithelium, it may occupy medial or more apical positions, pushing towards the glandular lumen (Figure 3A,D). The rough endoplasmic reticulum is widely disseminated throughout the cytoplasm in the form of densely packed, parallel-arranged cisternae, and is most commonly found at the base of the cell and in the perinuclear region (Figure 3B–E). Golgi systems are relatively small and uncommon while many thin and slender mitochondria are evenly distributed throughout the cytoplasm (Figure 3B–H) that is also characterized by the presence of several vesicles. Larger ones, containing irregular and electrondense material, are located medially in the cells (Figure 3D); in contrast, the smaller and more common electronlucid vesicles are mostly located in the apical region (Figure 3E,F,H), near the dense array of packed microvilli. Rare electrondense granules appear nearby and scattered between some of the microvilli (Figure 3F).

- Second pair of male accessory glands in *M. variabilis*. The second pair of male accessory glands has an outer muscular layer, made up of circular muscles on the inside and longitudinal outside, which encloses a glandular epithelium (Figure 4A,B). The columnar epithelial cells, roughly 25–30 μm high, are organized as a monolayer that exhibits pleats and ensuing involutions towards a large lumen (Figure 4A,C) that receives the secretory products appearing as dark amorphous bodies immersed in a lighter homogeneous matrix (Figure 4A–C). Contiguous cells are usually tightly adherent to each other and appressed to the basal lamina, however, in some regions of the epithelium they are partially detached from a 0.6–0.8 μm thick basal lamina, showing moderate folds and intercellular spaces in the baso-lateral plasma membrane (Figure 4B). A single ovoid nucleus of about 10 μm, with no indentations or lobes, is placed towards the center of the cell and is characterized by the presence of an evident central or eccentric nucleolus and sparse heterochromatin (Figure 4B–D). The rough endoplasmic reticulum is well-developed (Figure 4D–E) and located mostly near the nucleus, primarily in the form of parallel and flattened cisternae and, to a lesser extent, also in a vesiculate form with expanded cisternae (Figure 4F). Thin mitochondria (Figure 4C,E,F) are abundantly distributed throughout the cell, although they are more prevalent towards the apical region where stacks of long microvilli arise from the apical plasma membrane (Figure 4B,C,E). Several small vesicles with electronlucent contents and a few slightly electrondense secretory granules are disseminated throughout the cytoplasm (Figure 4C,E,F), while small and flattened Golgi complexes are rarely observed in these cells (Figure 4F).

- Third pair of male accessory glands in *M. variabilis*. The third pair of male accessory glands is composed of a comparatively thin layer of circularly and transversely oriented muscles, gently surrounding a monolayer epithelium that delimits a large lumen filled with a homogeneous secretion that appears as slightly electrondense fibrillar structures immersed in an almost lucid matrix (Figure 5A). The glandular cells, 5–8 μm high, are flattened, squamous or barely cuboidal and lay on a basal lamina 0.4–0.6 μm thick (Figure 5A–E). Their basal membrane is usually highly folded and only partially attached to the basal lamina, creating a developed labyrinthine network of intercellular spaces (Figure 5B,C). A basal and oval nucleus, 10–12 μm long, contains a large nucleolus and heterochromatin aggregates, and is surrounded by flattened stacks of rough endoplasmic reticulum and abundant Golgi complexes featuring small cisternae that are widespread in both the basal and apical regions of the cells (Figure 5C–E). The cytoplasm abounds in mitochondria and contains several small electronlucid vesicles, some of which contain a fine flocculent particulate (Figure 5B–F). A few electrondense secretory granules and rare multilamellar bodies are found basally in the cell (Figure 5D,E). Autophagic vacuoles containing irregular electrondense inclusions are also frequently present in the cytoplasm (Figure 5A, inset). Apically, the plasma membrane develops into widely spaced, long microvilli, some of which show a characteristic irregular, ampullaceous expansion with marked dark edges (Figure 5B,D).

- *Vasa deferentia* in *M. variabilis*. The glandular regions of the *vasa deferentia* have an outer tunica of developed striated muscle fibers that encloses a monolayered epithelium delimiting a central lumen that contains fibrillar secretions and spermatozoa bundles (Figure 6A).

Epithelial cells, 13–18 μm high, are cuboidal or slightly columnar and have a single oval nucleus 10 μm long with peripheral clumps of heterochromatin (Figure 6A,B). Nuclei are basally located in the cell, more or less along a single line within the epithelium (Figure 6A). Adjacent cells are usually tightly adherent to each other and firmly attached to a 0.3–0.45 μm thick basal lamina that interposes itself between the cells and the muscles (Figure 6B,C). Nevertheless, in some of its sections the epithelium develops fairly basolateral intercellular spaces (Figure 6C); in both cases, the plasma membranes exhibit a moderately sinuous course. Abundant mitochondria are mainly distributed apically and present themselves in quite different forms, most are moderate in size, thin and elongated, while a smaller number appear as more condensed, larger and with expanded or angular forms (Figure 6B–D). Rough endoplasmic reticulum membranes are present both as flattened, parallel-arranged cisternae and as expanded and swollen cisternae (Figure 6E). Many small electronlucent vesicles, some electrondense inclusions and rare secretory granules are dispersed in the cytoplasm (Figure 6C,F) while long overlapping microvilli can be observed on the apical surface of the cell facing the lumen (Figure 6B,D).

**Figure 7:**
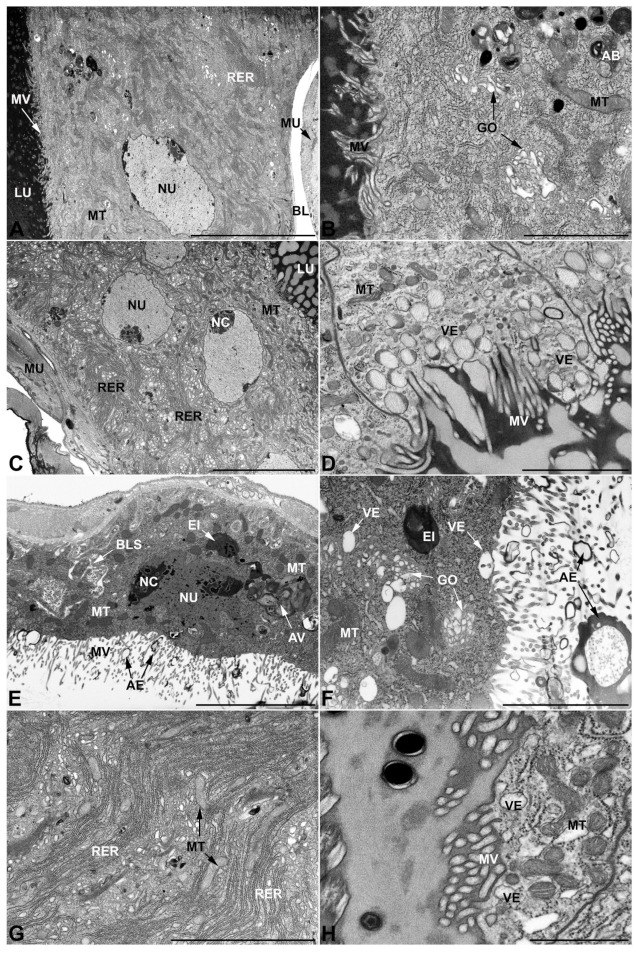
Ultrastructural features of *M. pusilla* and *M. flexuosa* male accessory glands and glandular region of the *vasa deferentia*. (**A**,**B**) First pair of male accessory glands of *M. pusilla* exhibiting a reduced level of pseudostratification. (**B**) Cytoplasm with small Golgi complexes, mitochondria and vacuolar bodies. (**C**,**D**) Second pair of male accessory glands of *M. flexuosa* with developed rough endoplasmic reticulum. Note the peculiar appearance of the content of the secretory vesicles located near the microvilli. (**E**,**F**) Third pair of male accessory glands of *M. flexuosa* exhibiting basal lacunar system, autophagic vacuoles, inclusions, numerous mitochondria, small Golgi complexes, minute electronlucid vesicles and microvilli with ampullaceous expansions. (**G**,**H**) *Vasa deferentia* of *M. pusilla.* Details of the rough endoplasmic reticulum (**G**) and apical microvillar region facing the lumen, displaying small vesicles. AB, autophagic body; AE, ampullaceous expansions; AV, autophagic vacuole; BL, basal lamina; BLS, basal lacunar system; EI, electrondense inclusion; GO, Golgi complex; LU, lumen; ML, multilamellar body; MU, muscles; MT, mitochondria; MV, microvilli; NC, nucleolus; NU, nucleus; RER, rough endoplasmic reticulum; VE, vesicles. Scale bars: (**A**) = 10 μm; (**B**) = 2 μm; (**C**) = 10 μm; (**D**) = 2 μm; (**E**) = 5 μm; (**F**) = 3 μm; (**G**) = 4 μm; (**H**) = 1 μm.

- Accessory glands and *vasa deferentia* in *M. flexuosa* and *M. pusilla*. In the two smaller congeneric species, *M. pusilla* and *M. flexuosa*, the cellular arrangement of the first pair of accessory glands appears similar to that observed in *M. variabilis*. Minor differences seem to be related to the smaller size of the gland, which present cells of diminished size (roughly 25–30 μm tall) with a reduced degree of pseudostratification (Figure 7A). The contents of the lumen are very similar in appearance (Figure 7A), as are the ultrastructural features of the cells, displaying abundant flattened endoplasmic reticulum, elongated mitochondria scattered throughout the cytoplasm, flattened Golgi complexes, minute lucent vesicles and larger vacuolar bodies containing dense structures (Figure 7A,B).

In addition, with regard to the second pair of glands, the differences appear to be restricted and relate mainly to the rough endoplasmic reticulum and the vesicles. The former, always well developed, shows a greater amount of dilated regions in comparison to what is observed in *M. variabilis* (Figure 7C), while the vesicles of *M. pusilla* display a distinctive content, which is presented as lamelliform structures of medium density immersed in an electronlucid matrix (Figure 7D).

The third pair of accessory glands show no meaningful differences within the genus *Mylabris*; in fact, also within *M. pusilla* and *M. flexuosa,* the cells display the main features and peculiarities previously described in *M. variabilis* (Figure 7E,F). These include the presence of basal intercellular spaces, ampullaceous expansions at the end of the microvilli, and the occurrence of a particulate secretion filling the lumen. Other ultrastructural features are also comparable: the nucleus is oval with a clearly visible nucleolus, and Golgi complexes are abundant and have small cisternae while the vesicles are minute and predominantly electronlucid.

The *vasa deferentia* of the different species of *Mylabris* investigated here are very similar to each other and are characterized by the presence of small electron lucent vesicles and by the extensive development of the endoplasmic reticulum, which is so widely distributed within the cell that it occupies a large part of the cytoplasm, either in the form of flattened cisternae or swollen vesicles (Figure 6G,H). Nevertheless, it is interesting to note that the high variability in mitochondrial morphology found in *M. variabilis* does not appear to be present in the two other species of *Mylabris* illustrated here, whose mitochondria do not show forms of increased size and angular appearance.

### 3.3. Ultrastructure of Accessory Glands and Vasa Deferentia in Lydus (Meloinae, Lyttini)

**Figure 8:**
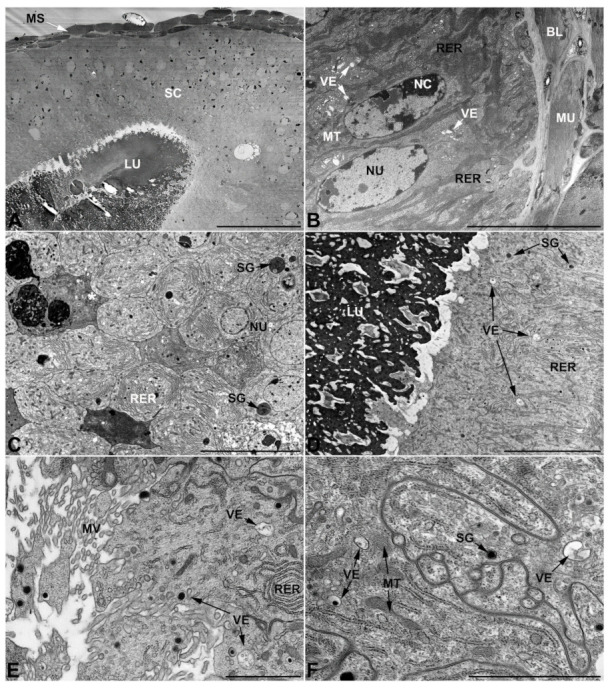
Ultrastructural features of the first pair of male accessory glands in *L. trimaculatus*. (**A**) Oblique section of the gland showing the high level of pseudostratification. (**B**) Longitudinal section illustrating the basal region of the cells, lying on the basal lamina. The cytoplasm contains regular oval nuclei, abundant rough endoplasmic reticulum and lucent vesicles. Note the presence of tracheae between the muscles. (**C**) Cross section of the gland in the medial region. Notable is the polygonal shape of the cells and the presence of secretory vesicles and granules. Some exhausted cells, indicated by asterisk, show a more electrondense cytoplasm. (**D**) Micrograph of the apical region of the cells where, in addition to the presence of vesicles and granules, the cell projections and their budding towards the lumen can be appreciated. (**E**) Detail of the apical region with microvilli and budding. (**F**) Close up showing membrane folding, mitochondria, vesicles and granules. BL, basal lamina; LU, lumen; MS, muscular sheath; MU, muscles; MT, mitochondria; MV, microvilli; NC, nucleolus; NU, nucleus; RER, rough endoplasmic reticulum; SC, secretory cells; SG, secretory granules; VE, vesicles. Scale bars: (**A**) = 50 μm; (**B**–**D**) = 10 μm; (**E**,**F**) = 2 μm.

In *L. trimaculatus*, the glands of the first pair are remarkably different from those observed in the genus *Mylabris*; not so much in their shape, which resembles that of *M. pusilla* glands (Figure 1), but in their cellular organization, since the cell monolayer, which constitutes the glands, shows a markedly higher degree of pseudostratification (Figure 8A–C). Another substantial difference concerns the apical microvillated region of the glandular cells, which presents exclusive budding areas that project towards the inner lumen of the gland (Figure 8D,E). These structures suggest the involvement of an apocrine secretion mechanism that is complementary to the exocytosis of the numerous vesicles characterizing these glands. Interestingly, such cellular processes protruding into the lumen were neither detected in *Mylabris* species nor in the other Meloidae species analyzed, in which the merocrine process appeared as the only detectable secretion mechanism in the first pair of accessory glands. Additional distinctive features concern the higher frequency of electrondense granules (Figure 8C,F), compared to what is observed in *Mylabris*, and a different appearance of the smaller vesicles, which are not filled exclusively with electronlucent material but also are frequently comprise of dark spheres immersed in the lucid matrix (Figure 8E,F). Conversely, even in *L. trimaculatus* the gland is made up by cells of a single type that are well appressed to each other, leading to their peculiar polygonal appearance (in cross section) (Figure 8B,C). Compared to *Mylabris* species, the flattened stacks of developed rough endoplasmic reticulum are equally abundant, elongated mitochondria are also similar in appearance and distribution while the nuclei appear more rounded and regular (Figure 8A–C).

**Figure 9:**
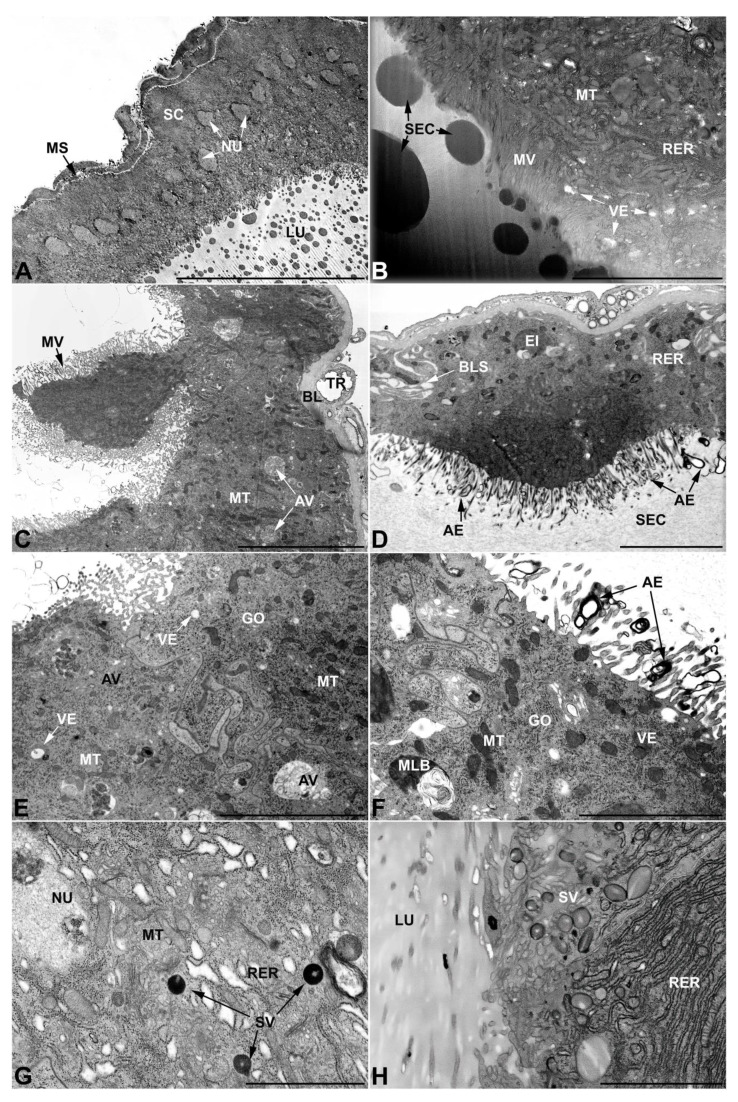
Ultrastructural features of *L. trimaculatus’* second and third pair of male accessory glands and glandular region of the *vasa deferentia.* (**A**) Transverse section of second pair gland showing monolayer of microvillated columnar cells and lumen containing dark spheroidal secretions immersed in a lighter matrix. (**B**) Apical region of the former bearing microvilli and exhibiting irregular vesicles and numerous mitochondria. (**C**–**F**) Sections of the gland of the third pair. (**C**) Note the increased height of the cells and the presence of autophagic vacuoles. (**D**) Microphotograph illustrating the basal lacunar system, numerous mitochondria, thin and elongated microvilli with expanded ampulla-like projections and particulate secretion within the lumen. (**E**,**F**) Details of mitochondria, vacuoles, multilamellar bodies, inclusions, small Golgi complex, microvilli and electronlucent vesicles. (**G**,**H**) Pictures of *vasa deferentia* cytoplasm showing typical, distinctive-looking secretory vesicles as well as rough endoplasmic reticulum in both flattened and expanded forms. AE, ampullaceous expansions; AV, autophagic vacuole; BL, basal lamina; BLS, basal lacunar system; EI, electrondense inclusion; GO, Golgi apparatus; LU, lumen; MLB, multilamellar body; MS, muscular sheath; MU, muscles; MT, mitochondria; MV, microvilli; NC, nucleolus; NU, nucleus; RER, rough endoplasmic reticulum; SC, secretory cells; SEC, secretory products; SV, secretory vesicles; TR, trachea; VE, vesicles. Scale bars: (**A**) = 40 μm; (**B**) = 5 μm; (**C**) = 10 μm; (**D**,**E**) = 5 μm; (**F**) = 3 μm; (**G**,**H**) = 2 μm.

Concerning the second pair of glands, it is worth noting that the secretion contained in the lumen has a very different appearance compared to the one occurring in the genus *Mylabris*, as the lumen is filled by an electron lucent matrix in which are immersed electrondense structures with rounded and regular contours that differ considerably from the irregularly shaped secretions observed in the genus *Mylabris* (Figure 9A). Additionally, in *L. trimaculatus*, mitochondria are abundantly distributed throughout the cytoplasm, with a preference for the apical region, near the microvilli, where several small vesicles with electronlucid content and an irregular and fringed shape can be observed (Figure 9B).

The glands of the third pair are very similar to those observed in the genus *Mylabris*, except that the cells appear more developed in height, having a cuboidal shape tending to columnar rather than squamous (Figure 9C). Otherwise, the cellular features are almost identical, resulting in a labyrinthine network of spaces at the base of the cell and ampullate expansions at the apical microvilli (Figure 9D–F). Similar to *Mylabris*, the cytoplasm shows abundant condensed mitochondria, diffused autophagic vacuoles, flattened rough endoplasmic reticulum, electrondense inclusions, and rare multilamellar bodies (Figure 9C–F). Similarities are also observed for the vesicles, which are small and electronlucent, and for the abundant Golgi complexes that occur in various regions of the cytoplasm in the form of rather small dictyosomes (Figure 9E,F).

*Vasa deferentia*, similarly to those observed in the genus *Mylabris*, are well defined by a highly developed and diffuse rough endoplasmic reticulum, which is found both in vesicular and flattened form (Figure 9G,H). The main difference detected within this tissue involves the vesicles’ appearance, with *L. trimaculatus* differing from the *Mylabris* species for the presence of specific vesicles containing electrondense material in which a rod-shaped structure of lower density is immersed (Figure 9H).

### 3.4. Ultrastructure of Accessory Glands and Vasa Deferentia in Epicauta (Meloinae, Epicautini)

**Figure 10:**
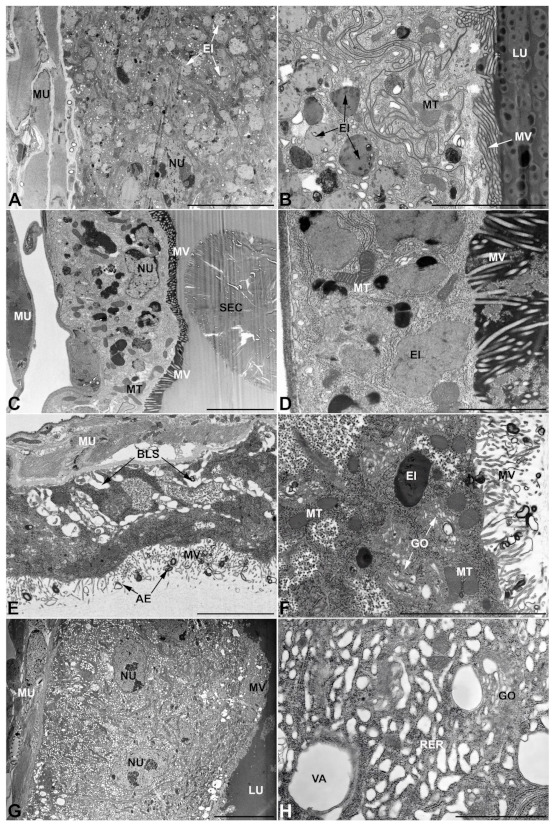
Ultrastructural features of *E. rufidorsum* male accessory glands and glandular region of the *vasa deferentia*. (**A**,**B**) First pair of glands. (**A**) Micrograph showing the pseudostratification of the monolayer, firmly adherent to the basal lamina, and the presence of well-developed muscles surrounding the epithelium. (**B**) Apical region of cells showing rounded inclusions and rough endoplasmic reticulum with enlarged forms. Note the appearance of the aggregates contained in the lumen. (**C**,**D**) Second pair of glands. (**C**) Cells have abundant mitochondria and show several cytoplasmic inclusions. Note the presence within the lumen of conspicuous laminar structures and a dark region near the microvilli. (**D**) Cytoplasm view showing rough endoplasmic reticulum, irregularly shaped inclusions with areas of increased electrondensity, mitochondria and microvilli. Note, also, the projections of the cell towards the lumen. (**E**,**F**) Gland of the third pair. (**E**) Cells exhibiting a developed lacunar basal system and bearing ampullaceous expansions at the level of the microvilli facing the lumen filled with particulate matter. (**F**) Apical region with microvilli bearing expansions. Note small Golgi complex, and inclusions of various sizes. (**G**,**H**) *Vasa deferentia* with high development of the wrinkled endoplasmic reticulum. Note the presence of vacuolar structures of increased size. AE, ampullaceous expansions; BLS, basal lacunar system; EI, electrondense inclusion; GO, Golgi complex; LU, lumen; MU, muscles; MT, mitochondria; MV, microvilli; NU, nucleus; RER, rough endoplasmic reticulum; SEC, secretory products; VA, vacuoles. Scale bars: (**A**) = 10 μm; (**B**) = 4 μm; (**C**) = 5 μm; (**D**) = 3 μm; (**E**,**F**) = 5 μm; (**G**) = 10 μm; (**H**) = 2 μm.

In *E. rufidorsum*, the glands of the first pair differ further from those described so far, in fact, while maintaining a pseudostratified structure its cells are characterized by the presence of high amounts of rounded inclusions of different electrondensities, uniformly scattered throughout the cytoplasm (Figure 10A,B). The rough endoplasmic reticulum also differs in appearance, occurring not only in the form of flattened stacks, as seen in the first pair of glands so far, but also in an expanded and vesicular form (Figure 10B). Moreover, the secretion contained in the lumen appears to be unusual and different from those previously illustrated, appearing as a collection of aggregates of variable electrondensity (Figure 10B).

The glands of the second pair also differ significantly from those presented earlier, starting from the substances that fill the glandular lumen, which appear as large structures with a laminated pattern and an oval profile (Figure 10C). In addition to these latter, which are immersed in an electronlucent matrix, the lumen is further defined by a more electrondense substance located near the apical region of the cell from which the microvilli arise (Figure 10D). Finally, among the diverging features found in these cells, it is interesting to note both the presence of large inclusions as well as the fact that, in the vicinity of the microvilli, small projections shedding towards the glandular lumen can be observed (Figure 10D).

Unlike the accessory glands of the first and second pairs, the accessory glands of the third pair (Figure 10E,F) do not differ from those of the species illustrated in the previous paragraphs. The secretion contained in the lumen has the same particulate appearance, the cells are squamous, show a lacunar system at their base and apical ampullary expansion at the level of the microvilli. Golgi complexes are abundant throughout the cell and are characterized by small cisternae, mitochondria are frequent and well dispersed in the cytoplasm, and the secretory vesicles are minute and electronlucid (Figure 10F). Several inclusions and electrondense granules are also present in these cells.

As for the glandular part of *vasa deferentia*, their cells are similar to those previously described by the strong presence of rough endoplasmic reticulum containing a large number of enlarged vesicles (Figure 10G,H). Note as a distinctive feature, which seems to be characteristic of this species, the presence of vacuolar structures of a generous size that accompany smaller vesicular structures, both filled with translucent substances (Figure 10H).

### 3.5. Ultrastructure of Accessory Glands and Vasa Deferentia in Cerocoma (Meloinae, Cerocomini)

**Figure 11:**
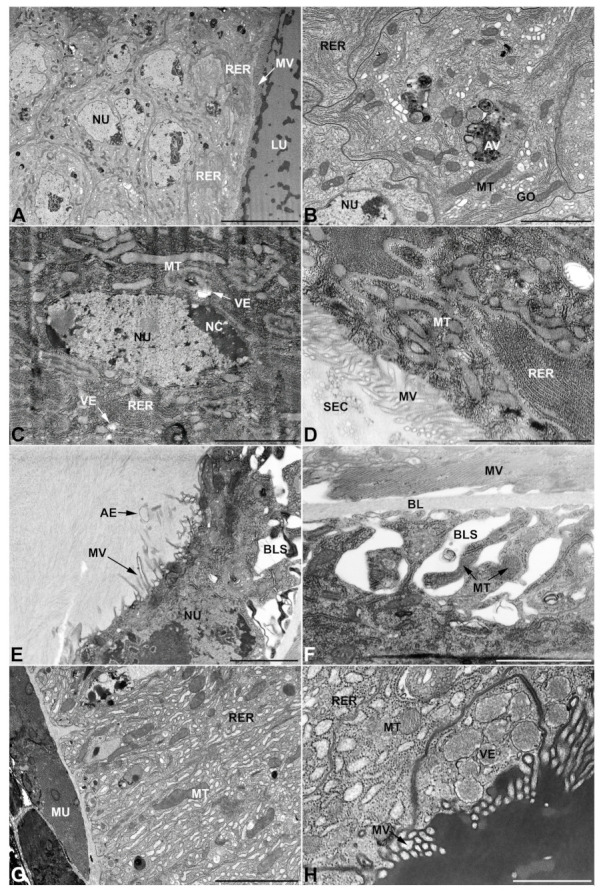
Ultrastructural features of *C. schreberi* male accessory glands and glandular region of the *vasa deferentia*. (**A**,**B**) Gland of the first pair. (**A**) Oblique section of the accessory gland showing the cells contours appearing moderately sinuous and only slightly polygonal. Note the lumen filled with electrondense irregular secretions immersed in a uniform matrix of lesser electrondensity. (**B**) Detail of cells of the first pair of glands showing elongated mitochondria, flattened rough endoplasmic reticulum, abundant Golgi complex, electron lucent vesicles and autophagic vacuoles. (**C**,**D**) Micrographs of the second pair of accessory glands displaying the ovoid nucleus surrounded by mitochondria and a rough reticulum. (**C**) The apical region with numerous mitochondria near the thin microvilli that face a glandular lumen with a pale and uniform content (**D**). (**E**) Gland of the third pair exhibiting particulate secretion stored in the lumen, slender apical microvilli with scarce ampullaceous expansion and developed basal lacunar system. (**F**) Close up of the basal lacunar system of the third pair of glands, note the presence of mitochondria in proximity to the lacunar spaces and invaginations of the basal membrane. (**G**,**H**) Glandular part of *vasa deferentia*. (**G**) Transverse section showing muscles, basal lamina and the basal region of the cells rich in rough endoplasmic reticulum with swollen cisternae and some electrondense granule. (**H**) Apical region with the peculiar secretory vesicles approaching to the short microvilli. AE, ampullaceous expansions; AV, autophagic vacuole; BL, basal lamina; BLS, basal lacunar system; GO, Golgi apparatus; LU, lumen; MU, muscles; MT, mitochondria; MV, microvilli; NC, nucleolus; NU, nucleus; RER, rough endoplasmic reticulum; SEC, secretory products; VE, vesicles. Scale bars: (**A**) = 10 μm; (**B**–**F**) = 2 μm; (**G**) = 5 μm; (**H**) = 1 μm.

Cells of the first pair of glands retain the pseudostratified organization that is common to all the species illustrated so far, but in *C. schreberi* the lateral membranes of these cells appear slightly less straight and more sinuous, leading to a less polygonal appearance of their borders (Figure 11A,B). In addition to this difference is the presence of an apparently increased number of Golgi complexes, as well as the presence of structures similar to the autophagic vacuoles often found in third-pair glands and containing organelle remnants (Figure 11B).

The glands of the second pair (Figure 11C,D) have small irregular electronlucent vesicles, as seen in *L. trimaculatus*, but differ from the latter by having a lumen filled with a uniformly pale secretion. Otherwise, the cells show common features, such as a well-developed rough endoplasmic reticulum located close to the nucleus as abundant stacks.

The glands of the third pair (Figure 11E,F) show no differences from those analyzed in other blister beetles, except for a diminished presence of ampullary expansions. The network of spaces at the base of the cells is present, and the organelle structure is also similar to that seen in other species with very small Golgi complexes and an endoplasmic reticulum exclusively represented in its flattened form.

Furthermore, in *C. schreberi* the *vas deferens* (Figure 11G,H) show an copious amount of rough endoplasmic reticulum with a high percentage of expanded and vascularized cisternae, an abundance of well-developed mitochondria, and cytoplasmic inclusions that are similar to those observed in *M. variabilis* (Figure 11G). However, the *vas deferens* of this species differs in the presence of unique secretory vesicles, having irregular borders and containing a granular substance of medium density (Figure 11H).

### 3.6. Ultrastructure of Accessory Glands and Vasa Deferentia in Zonitis (Nemognatinae, Nemognatini)

**Figure 12:**
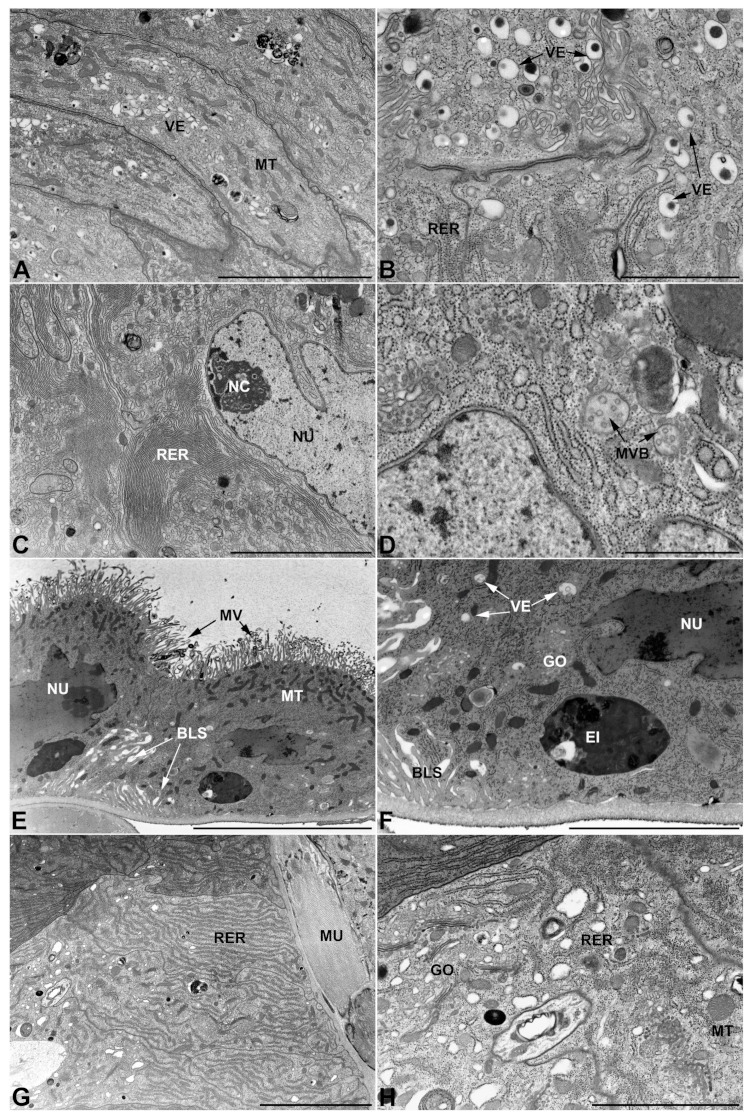
Ultrastructural features of *Z. immaculata* male accessory glands and glandular region of the *vasa deferentia*. (**A**,**B**) Gland of the first pair. (**A**) Polygonal appearance of cells in oblique section. The cytoplasm shows numerous vesicles. (**B**) Secretory vesicles in the apical region, near the microvilli. (**C**,**D**) Second pair of glands. (**C**) Abundant rough endoplasmic reticulum in proximity to a nucleus. (**D**) Apparent structures as multivesicular bodies. (**E**,**F**) Glands of the third pair. (**E**) Cells showing a basal lacunar system and numerous mitochondria clustered towards the apical region, near the microvilli. (**F**) Close up of the previous image, note a large inclusion. (**G**,**H**) *Vasa deferentia* with mostly flattened rough endoplasmic reticulum. BLS, basal lacunar system; EI, electrondense inclusion; GO, Golgi complex; MT, mitochondria; MU, muscles; MVB, multivesicular body; MV, microvilli; NC, nucleolus; NU, nucleus; RER, rough endoplasmic reticulum; VE, vesicles. Scale bars: (**A**) = 5 μm; (**B**) = 2 μm; (**C**) = 4 μm; (**D**) = 1 μm; (**E**) = 10 μm; (**F**) = 4 μm; (**G**) = 5 μm; (**H**) = 2 μm.

In *Z. immaculata* the glands of the first pair retain the polygonal appearance already seen in other species (Figure 12A) along with many other features typical of the first pair of glands, but differ from the glands of other blister beetles for the presence of numerous lucent vesicles containing small spherical structures that are strongly electrondense (Figure 12B).

Although it shares common features with other previously examined species, the second pair of glands exhibits some interesting peculiarities, such as the high development of the rough endoplasmic reticulum in the form of both abundant and closely arranged stacks, and swollen vesicles of a modest size (Figure 12C,D). Among the unique features of this tissue, we also find the presence of structures appearing as multi-vesicular bodies that have not been detected in glandular tissues of other species.

Even in *Z. immaculata*, as seen in the previous species, the third pair of glands does not show substantial differences, in fact glands display cells with a widespread basal labyrinthine network of spaces and apical ampullary expansions towards the microvilli (Figure 12E). The cells are also characterized by abundant mitochondria, very small dictyosomes, electrondense inclusions and small transparent secretory vesicles (Figure 12F).

With regard to *vasa deferentia* it is interesting to note that, although the rough endoplasmic reticulum is highly developed throughout the cytoplasm, the number of flattened stacks of rough endoplasmic reticulum is much higher than in the other species, with the flattened form representing the most abundant portion compared to the vesicular one, which is particularly reduced (Figure 12G,H).

## 4. Discussion

The present study describes in detail the ultrastructural features of the male accessory glands and the glandular region of the *vasa deferentia* in seven species of Meloidae, which belong to three distinct tribes of the subfamily Meloinae (Mylabrini, Lyttini, Cerocomini) and to one tribe of the subfamily Nemognathinae (Nemognathini). These analyses allowed to evaluate the degree of variability of these glandular tissues, revealing the presence of several differences as well as conserved characteristics at various levels.

The male reproductive systems of the Meloidae species analyzed here show a common organization involving a pair of large *vasa deferentia* and three pairs of accessory glands of mesodermal origin, and releasing their contents at the top of an ejaculatory duct of ectodermal origin. This configuration, with glandular *vasa deferentia* and three pairs of accessory glands, is shared by most of Meloidae species, as evidenced by the monograph of Beauregard [22] and the multiple works of Gupta [47,48,49,50] that presented extensive information and illustrations on the general structure of both male and female reproductive systems. Only a limited number of notable exceptions to this “three glands pattern” have been reported for the following species: *Horia debyi* (Fairmaire, 1885) (as *Cissites testaceus*) (Nemognathinae, Horiini), having a single pair of accessory glands [55]; *Hycleus phaleratus* Pallas, 1781 (as *Mylabris*) (Meloinae, Mylabrini), having two pairs [56]; and *Epicauta rufidorsum* Goeze, 1777 (as *Epicauta verticalis*) (Meloinae, Epicautini), having four pairs of accessory glands [22]. Our results confirm Gupta’s observations for the genus *Epicauta* regarding the presence of three pairs of accessory glands [47], while disagreeing with Beauregard’s observation of the presence of a fourth pair of accessory glands [22]. The case of *E. rufidorsum* deserves further investigation to assess whether we are in the presence of an interpopulation difference.

From a gross morphological perspective, the shape of the first pair of glands in the species analyzed is highly variable, with forms ranging from simple tubular structures (as in *E. rufidorsum*, *Z. immaculata*) to strongly spiraled ones (as in *C. schreberi*, *M. pusilla*, *M. flexuosa* or *L. trimaculatus*), as well as some intermediate conditions with more or less pronounced folds, curvatures, or coils (as in *M. variabilis*). Shape patterns found in our examined species are consistent with the previously published literature data from Gupta [47,48,49,50], Beauregard [22], and Gerber and colleagues [51,52]. The previous literature also indicates that spiral glands seem to be among the most common, while the tubular form is much less frequent and is limited to rare specific cases. The presence of very different forms even within the same genus, as can be seen in the three species of *Mylabris* analyzed here, and the extensive presence of a first pair of spiral accessory glands that can be found in almost all tribes of Meloidae [22,47,48,51], clearly shows that accessory gland shape does not seem to trace any phylogenetic relationships.

In the investigated species, the second pair of glands has always a tubular shape and is usually not particularly developed in length, except for the glands of *M. variabilis*. The comparison with previous data [22,47,48,51] shows that both the tubular shape as well as the moderate length are the most common characteristics in Meloidae, but the presence of elongated structures is not exceedingly rare as it can be found in other *Mylabris* species and in species belonging to other genera, such as *Meloe* Linnaeus, 1758, *Epicauta* or *Zonitis* Fabricius, 1775 [22,47].

The glands of the third pair, on the other hand, appeared remarkably similar among the analyzed species, being much more delicate and longer than the other pairs and exhibiting an irregular and sinuous shape that causes them to become entangled with the neighboring tissues. This tendency towards uniformity in the gland’s general structure, within the family Meloidae, seems to be confirmed by the literature data referring to other blister beetle species [22,47,48,49,50,51].

In the analyzed species, the gross morphology of *vasa deferentia*, characterized by an enlarged volume and the presence of a large and expanded glandular region, also appeared very similar, following a common pattern throughout the family [22,47,48,49,50,51].

The ultrastructural analysis has shown that in all the studied species, the different pairs of accessory glands and the *vas deferens* have several common features. They all have a mesodermal derivation and are always enclosed by a muscular sheath with differently oriented muscles, which facilitate the mobilization of the secretions contained in their large lumens. All these glands consist of a monostratified epithelium with cells varying from squamous, typically observed in the third pair of glands, to columnar, usually present in the first pair. Only the first pair of glands slightly differs from the others in its cellular organization, being the only one that invariably displays a pseudostratified epithelium in all the analyzed species, a feature absent in the other pairs of glands.

The different types of glandular tissues described here share many of the ultrastructural features that are typical of cells involved in active protein synthesis, such as an abundance of rough endoplasmic reticulum, many mitochondria, the presence of apical membrane with microvilli and a large number of vesicles and secretory granules [57,58,59,60,61]. Despite these similarities, each pair of glands seems to be involved in the production of a unique and specific substance, as suggested both by the different appearance of the secretions contained in the lumen and by the fact that the glandular epithelium is composed of a single cell type, each of which has its own ultrastructural peculiarities. It is also interesting to note that the presence of only one type of cell in the accessory glands of the Meloidae analyzed here represents a markedly different pattern from that found in other beetles with only one or two pairs of accessory glands, in which several types of secretory cells are found together in the same gland, each specialized in the production of a different secretion that is discharged into a common lumen [62,63,64,65,66].

By comparatively analyzing the ultrastructure of each pair of accessory glands in the examined species and with reference to the data available for *M. proscarabaeus* [53], we can note a high level of variability for many of these structures.

The glands of the first pair have in all the considered species a pseudostratified epithelium with polygonal cells, except for *C. schreberi* where the cells are less packed and have membranes with more rounded borders. On the other hand, among the studied species, there is a moderate variability in the appearance of the secretory vesicles, often small and electronlucid, but occasionally with dense spherical bodies inside. Such structures can be found in species considered distant (e.g., *L. trimaculatus* and *Z. immaculata*) and their presence does not seem to correspond to a close phylogenetic relationship [4]. Perhaps one of the most interesting differences concerns the type of secretion found in the examined species, among which only *L. trimaculatus* showed an apocrine type of secretion with the release of part of the cell within the lumen. In all other cases, the only means of secretion detected was via a merocrine one by simple exocytosis of the vesicles. Another interesting difference can be observed in *E. rufidorsum*, which is the only species to present a high number of cytoplasmic inclusions at the level of this gland, highlighting how the cytoplasmic features can vary between different species. Finally, the appearance of the substances contained in the lumen is often very different from one species to another.

The glands of the second pair show an even greater variability starting from the appearance of the secretion contained in the lumen, which ranges from an irregular shape, as in the genus *Mylabris*, spheroidal, as in *Lydus* Dejean 1821, to laminated structures, as seen in *Epicauta*. It is also interesting to underline the high variability in the characteristics of the secretory vesicles, even within the same genus, as observed with the species *M. pusilla*, which presents vesicles containing lamellar structures that were not reported in any other of the congeneric species investigated. Finally, it is noteworthy that also in the previously described glands of *M. proscarabaeus* [51], the vesicles found in the second pair of glands have a double membrane and contain particulate matter, making them strongly different from the electronlucid vesicles found in the other pairs of glands.

The *vasa deferentia*, on the other hand, are rather conserved and have cells that are easily distinguishable by the high development of the rough endoplasmic reticulum containing, usually, many swollen cisternae. However, these tissues are not free from ultrastructural peculiarities typical for each species. In this regard, it is worth noting the presence of secretory vesicles with a typical appearance and characteristic of a given species, as observed in the cases of *L. trimaculatus* or *C. schreberi*.

Finally, it is surprising to note that in contrast to the high variability among the species considered, the glands of the third pair are remarkably consistent from an ultrastructural point of view. It can be noted that the secretion filling the lumen is always identical and consists of a finely particulate substance. Several cellular characteristics are also recurrent, such as the presence of a lacunar system at the base of the cells, suggesting exchanges with the hemolymph, or the presence of particular ampulla-like expansions at the microvilli level. These could indicate an apocrine release of secretions in addition to the merocrine release of small electronlucid vesicles, which are also invariably encountered in all the species studied. The presence of numerous cytoplasmic inclusions also seems to be constant among the species analyzed, as is the frequent occurrence of autophagic vacuoles and multilamellar bodies that could indicate a high turnover of organelles.

Such an observed invariant ultrastructure of the third pair gland cells was also observed in *M. proscarabaeus* [53], and probably is widespread among the whole Meloidae. We can hypothesize that this similarity in the third pair, compared to the great variability of the other pairs of glands, is attributed to a strongly conserved function that is not influenced by the different reproductive strategies encountered in this family [23], nor by those differences in the female genital tract that may exert a strong influence on the characteristics of the spermatophores produced by the male accessory glands [47].

As already proposed for *M. proscarabaeus*, the constant presence of a basal lacunar system together with the highly conserved ultrastructural features suggests that the third pair of glands in Meloidae might have a key role in the management of cantharidin, as it is possible that these glands absorb the terpene contained in the hemolymph in order to concentrate it in their lumen. Although the importance of accessory glands in cantharidin production has been diminished by transcriptomics findings [44,45,67], it is extremely interesting that these glands are capable of sustaining high concentrations of terpene. Moreover, considering that the mechanisms involved in lowering the toxicity of this chemical remain unknown, male accessory glands still represent an extremely interesting tissue whose study could provide new information on the biochemical processes related to cantharidin in blister beetles.

## Data Availability

All microscopy data are available in the Interdepartmental Laboratory of Electron Microscopy (L.I.M.E.), Department of Science, University Roma Tre, Italy.
